# Characterizing patients issued DNR orders who are ultimately discharged alive: a retrospective observational study in Japan

**DOI:** 10.1186/s12904-020-00588-z

**Published:** 2020-06-09

**Authors:** Tomoari Mori, Katsumi Mori, Eisuke Nakazawa, Seiji Bito, Yoshiyuki Takimoto, Akira Akabayashi

**Affiliations:** 1 Department of Biomedical Ethics, School of Public Health, The University of Tokyo Graduate School of Medicine, 7-3-1 Hongo, Bunkyo-ku, Tokyo, 113-0033 Japan; 2 Division of Clinical Epidemiology, National Hospital Organization, Tokyo Medical Center, 2-5-1 Higashigaoka, Meguro-ku, Tokyo, 152-0892 Japan; 3 Division of Medical Ethics, Department of Population Health, New York University School of Medicine, 227 East, 30th Street, New York, NY 10016 USA

**Keywords:** Do not resuscitate (DNR) order, Retrospective study, Outcome, Timing, General hospital, Japan

## Abstract

**Background:**

The present study aimed to characterize factors associated with patients issued DNR orders during hospitalization who are discharged alive without any instruction orders by physicians regarding end-of-life treatment, with a focus on the timing of DNR order issuance.

**Methods:**

In total, 2997 DNR cases from all 61,037 patients aged ≥20 years admitted to a representative general hospital in Tokyo were extracted and divided into two groups by patient hospital release status (discharged alive/deceased). Study items included age, sex, disease type (non-cancer/cancer), hospital department (internal medicine/others), timing of DNR order issuance, implementation (or not) of life-sustaining treatment (LST) or the presence of any restrictions on LST and hospital length of stay. We conducted multiple logistic regression analysis, setting hospital release status as the dependent variable and each above study item as explanatory variables.

**Results:**

DNR orders were issued at a rate of 4.9%. The analysis revealed that patients with a DNR who were ultimately discharged alive were statistically more likely to be those for whom DNR orders are issued early after admission (adjusted odds ratio: AOR, 13.7), non-cancer patients (AOR, 3.4), internal medicine department patients (AOR, 1.63), females (AOR, 1.34), and elderly (aged ≥85 years; AOR, 1.02); these patients were also less likely to be receiving LST (AOR, 0.36).

**Conclusions:**

By focusing on those with DNR orders who were ultimately discharged alive, we discovered that these patients were likely to have DNR orders issued early after admission, and that they were more likely to be elderly, female, non-cancer patients, or those in internal medicine departments. Further examination of these data may help to elucidate why these particular DNR-related characteristics (including socio-economic and cultural factors) are evident in patients who end up being discharged alive.

## Background

The decision to withhold or withdraw a life sustaining treatment at the terminal phase of life has significant meaning. The Do-Not-Resuscitate (DNR) order is one example of such instructions with treatment restriction as their objective. According to guidelines of the American Medical Association, DNR orders are issued according to patient wishes and medical futility issues such as prognosis, with the aim of avoiding cardiopulmonary resuscitation (CPR) [1]. In Japan as well, the guideline of the Ministry of Health, Labour and Welfare recommends respecting the wishes of patients. However, this has yet to be fully realized in the Japanese context for several reasons, partly because advance directives are not legally binding.

One primary research question that has emerged through our experiences is: Why are there so many patients with DNR orders who are ultimately discharged without fatal conditions? These patients do not have Physician Orders for Life-sustaining Treatment (POLST) [2, 3], as these have not been legally introduced in Japan. Moreover, since this concept has not been widely introduced to Japan’s medical community, no patient in Japan has an official POLST.

In our observations of these patients, we have noted that they tend to have DNR orders issued soon after admission.

Previous studies [4, 5] have pointed out that if DNR orders are issued soon after onset when the prognosis is still unclear, other necessary treatments aside from CPR may also be restricted, resulting in a treatment course that is not beneficial. Thus, the timing of DNR order issuance is an important aspect to explore, as well as the potential of DNR orders to improve the patient’s quality of life. A DNR order is not always detrimental to patient experience.

Accordingly, the present study was designed to examine how patient hospital release status (discharged alive/deceased) is associated with other variables including the timing of DNR issuance, in order to better understand those patients with DNR orders who are discharged alive. The first step of this retrospective study was to identify subjects issued a DNR from patient records. Then, dividing them into two groups (discharged/deceased), we analyzed the discharged patients’ timing of DNR order issuance as well as several attributes available from their medical records, by comparing them to those of patients who died in the hospital (deceased). The present study thereby aimed to characterize factors associated with patients issued DNR orders during hospitalization who are discharged alive without any instruction orders by physicians regarding end-of-life treatment, with a focus on the timing of DNR order issuance. The findings obtained in this exploratory descriptive study are expected to serve as a basis for further discussions on the role of DNR orders in the clinical context.

## Methods

### Subjects

Study subjects included all adult patients (20 years and older; *n* = 61,037) admitted to a representative general hospital in the metropolitan Tokyo area with 780 beds between January 1, 2009 and December 31, 2012, and discharged by December 31, 2013. Patients who were hospitalized multiple times during this period were treated as separate cases per hospitalization.

### Study items

From the electronic medical records, we extracted basic information on patients, physician instructions, and procedure slips/vouchers for use in the study. All data were managed with Microsoft Excel software and were fully anonymized before use. Information on patient age, sex, disease type (cancer/non-cancer), department (internal/other), hospital release status (discharged/deceased), hospitalization period, and presence/absence of DNR order. If a DNR order was issued, the timing (how many days after admission), invasive life sustaining treatment (LST) (performed or not), and instructions to restrict invasive LST (presence/absence) were also extracted. The present study defined DNR as “instruction to avoid chest compressions (including airway management) upon cardiopulmonary arrest” and invasive LST as “intubation/ventilator use, dialysis, and blood-related product transfusion.”

### Extraction of search words for DNR and LST

From the physician’s instructions described above, we conducted an automated search of the Microsoft Excel database using DNR-related keywords: ‘DNR,’ ‘Do-Not-Attempt-Resuscitation (DNAR),’ ‘cardiopulmonary arrest (CPA),’ ‘CPR,’ ‘cardiac massage,’ and ‘chest compressions.’ For LST, search terms were ‘intubation,’ ‘ventilator use,’ ‘dialysis,’ and ‘blood-related product transfusion.’ Two independent coders with over 10 years of experience working in acute care medicine and who have graduate-level research experience read the relevant portions and identified the cases for which these terms were used appropriately with regard to DNR orders. In cases for which patient medical records had the terms ‘DNR,’ ‘DNAR,’ ‘No CPR,’ or when the written content implied avoidance of cardiac massage and chest compressions, those were considered to have fulfilled the definition of a DNR order for the purposes of the present study. Inter-coder discrepancies were resolved by further evaluation until a consensus was met. Of all cases targeted by the study, those deemed to have a DNR order issued were subject to analysis. We extracted words and phrases related to LST in the same manner.

### Analysis

First, patients with DNR orders were extracted from among all hospitalized patients, tallied, and classified by hospital release status (discharged/deceased), and fundamental statistics were compared for all study items. Next, discharged/deceased DNR cases were further stratified into three categories according to the timing of DNR order issuance to examine the relationship with each study item. Finally, multiple logistic regression analysis was performed to evaluate quantitatively the relationship between DNR cases with “discharged alive” status and each study item.

With regard to the timing of DNR order issuance, subjects were operationally categorized as follows: 1) “late” if the DNR was ordered within 3 days before the patient was discharged from the hospital; 2) “early” if the DNR was ordered within 3 days of hospital admission; and 3) “mid-term” for all others. Other study items were sorted by the timing of DNR order issuance (early, mid-term, and late) and compared between discharged and deceased groups. The Wilcoxon rank-sum test was used to analyze age and hospital length of stay. All other items were analyzed by Fisher’s exact probability test. Holm’s method was used to adjust for multiplicity.

Following this, hospital release status (discharged/deceased) was set as the dependent variable and all other study items as explanatory variables in order to calculate the adjusted odds ratio (AOR) for each item in multiple logistic regression analyses.

In order to avoid using an arbitrary cut-off of 3 days as a dividing line for the timing of DNR order issuance, separate logistic regression analyses were performed using days 1, 2, 4, and 5 as criteria.

Regarding the ‘early’ to ‘late’ definition, two analyses were conducted. The first used the following criteria as above: 1) If a DNR order is issued within 3 days before discharge, the patient is categorized into the ‘late’ group. For the remaining patients with DNR orders: 2) If a DNR order is issued within 3 days after admission, the patient is categorized into the ‘early’ group; and 3) all remaining patients are categorized into the ‘middle’ group (late priority criteria). Based on this grouping, if a patient dies 4 days after admission, and the DNR order was issued on day 2, then this patient would be categorized into the late group, even though the DNR order was issued on day 2. As this could be confusing, another analysis was conducted using the following criteria: 1) If a DNR order is issued within 3 days after admission, the patient is categorized into the ‘early’ group.

For the remaining patients with DNR orders: 2) If a DNR order is issued within 3 days before discharge, the patient is categorized into the ‘late’ group, and the remaining patients are categorized into the ‘middle’ group (early priority criteria).

All statistical analyses were performed using SAS version 9.4 (SAS Institute Inc., Cary, NC, USA). *P* < 0.05 was considered statistically significant.

### Ethical considerations

The present study was approved by the Ethics Committees of the University of Tokyo (Review No. 10321) and the National Hospital Organization Tokyo Medical Center (No. R13–063). Permissions were needed to access the data and the committees granted to do so. The committees approved opt-out consent by patients. The study protocol was provided on the website of the National Hospital Organization Tokyo Medical Center, and patients who wished to opt-out had the opportunity to do so. Patients and family who did not want to be used the data could contact the researcher, and their data were not used for analysis. Those who did not contact the researcher were assumed to have consented to participate in the study.

## Results

**Fig. 1:**
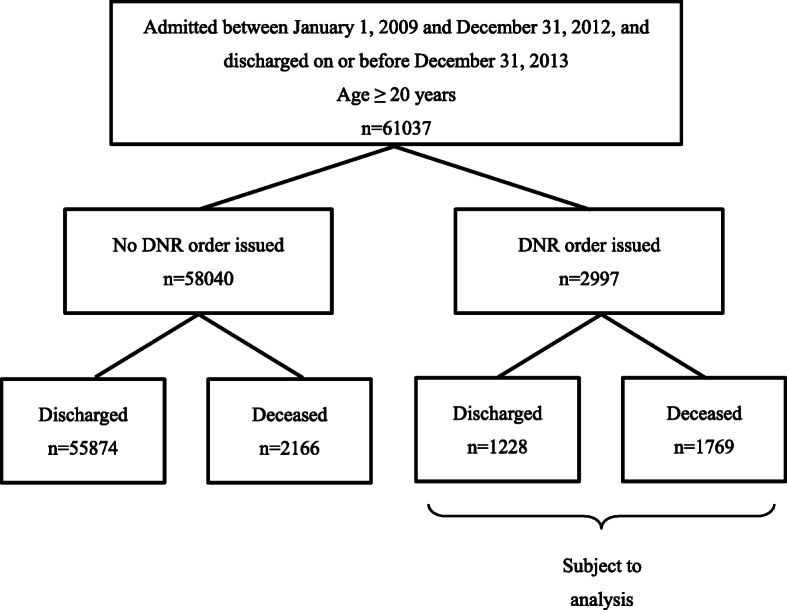
Study case selection process

Figure 1 shows a schematic of the subject selection process. Of the 61,037 subjects studied, 2997 (4.9%) had DNR orders, of whom 1228 (41.0%) were discharged (alive) and 1769 (45.0%) died during hospitalization.

Each item is displayed in Table 1 according to patient hospital release status (discharged alive/deceased). When patients were discharged alive, DNR orders were placed earlier (9.0 days vs 21.7 days) despite longer hospitalization (55.8 days vs 42.6 days). Those discharged alive were also likely to be older (84.4 y/o vs 77.4 y/o), female (59.4% vs 43.9%), internal medicine patients (91.3% vs 77.3%), had a non-cancerous condition (81.4% vs 51.3%), and received many invasive LST restriction orders (67.8% vs 57.9%).

**Table 1:** Simple tally of study variables for all study subjects and those with DNR orders as stratified by hospital release status (discharged or deceased)

	All study subjects		DNR order issued					
			All		Discharged		Deceased	
Patients, number	61,037		2997		1228		1769	
Age, years	64.1	(18.7)	80.3	(12.2)	84.4	(10.3)	77.4	(12.5)
Patients aged ≥85 years	6839	(11.2%)	1286	(42.9%)	738	(60.1%)	548	(31.0%)
Female patients	32,426	(53.1%)	1506	(50.3%)	730	(59.4%)	776	(43.9%)
Non-cancer patients	47,010	(77.0%)	1907	(63.6%)	999	(81.4%)	908	(51.3%)
Internal medicine patients	25,586	(41.9%)	2488	(83.0%)	1121	(91.3%)	1367	(77.3%)
Patients who received invasive LST	7935	(13.0%)	1073	(35.8%)	277	(22.6%)	796	(45.0%)
Patients with invasive LST restrictions	1941	(3.2%)	1858	(62.0%)	833	(67.8%)	1025	(57.9%)
Time until DNR issuance, days			16.5	(35.5)	9.0	(24.2)	21.7	(40.8)
Hospital length of stay, days	16.5	(31.1)	48.0	(65.0)	55.8	(76.3)	42.6	(55.3)
Deceased	3935	(6.4%)	1769	(59.0%)				

Age, time until DNR issuance, hospital length of stay: mean (standard deviation)

When the two groups were further stratified by the timing of DNR order issuance (early, mid-term, late) (Table 2), 63.6% (781/1228) of patients in the discharged alive group were issued DNR orders within 3 days after admission (‘early’ group), while 2.5% (31/1228) were issued DNR orders within 3 days before discharge (‘late’ group). In the deceased group, 29.7% (525/1769) were categorized as the ‘early’ group, and 24.8% (438/1769) as the ‘late’ group.

**Table 2:** Study variables by hospital release status (discharged or deceased)

	Discharged		Deceased		Comparison of Discharged vs Deceased ^†^
Timing of DNR order: Early					
Patients, number	781		525		
Age, years	85.8	(10.1)	81.5	(11.5)	**
Patients aged ≥85 years	519	(66.5)	236	(45.0)	**
Female patients	501	(64.1)	266	(50.7)	**
Non-cancer patients	661	(84.6)	293	(55.8)	**
Internal medicine patients	736	(94.2)	443	(84.4)	**
Patients who received invasive LST	125	(16.0)	165	(31.4)	**
Patients with restrictions on invasive LST	540	(69.1)	337	(64.2)	ns
Hospital length of stay, days	38.9	(42.3)	29.5	(32.7)	**
Timing of DNR order: Mid-term					
Patients, number	416		806		
Age, years	81.7	(10.1)	75.7	(12.0)	**
Patients aged ≥85 years	199	(47.8)	185	(23.0)	**
Female patients	213	(51.2)	328	(40.7)	**
Non-cancer patients	316	(76.0)	355	(44.0)	**
Internal medicine patients	359	(86.3)	593	(73.6)	**
Patients who received invasive LST	150	(36.1)	398	(49.4)	**
Patients with restrictions on invasive LST	280	(67.3)	443	(55.0)	**
Hospital length of stay, days	90.9	(108.9)	62.5	(67.9)	**
Timing of DNR order: Late					
Patients, number	31		438		
Age, years	82.9	(13.1)	75.7	(13.4)	**
Patients aged ≥85 years	20	(64.5)	127	(29.0)	**
Female patients	16	(51.6)	182	(41.6)	ns
Non-cancer patients	22	(71.0)	260	(59.4)	ns
Internal medicine patients	26	(83.9)	331	(75.6)	ns
Patients who received invasive LST	2	(6.5)	233	(53.2)	**
Patients with restrictions on invasive LST	13	(41.9)	245	(55.9)	ns
Hospital length of stay, days	9.3	(16.8)	21.7	(35.4)	ns

DNR: do not resuscitate, LST: life-sustaining treatment, IC: informed consent. Age and hospital length of stay: mean (standard deviation). †) Age and hospital length of stay were analyzed using the Wilcoxon rank-sum test, while other variables were analyzed using Fisher’s exact probability test. Holm’s method was used to adjust for multiplicity. **: *p* < 0.01, ns: not significant

The differences observed in the distribution of age, sex, disease type, department, and provision of invasive LST between the discharged and deceased groups were robust and consistent, even after DNR cases were divided according to timing (Table 2). However, due to the small number of patients in the ‘late’ DNR group, no significant differences were observed in sex, disease type, and department.

Compared to the ‘early’ group, the proportion of cancer patients and the frequency of invasive LST implementation were increased in the ‘late’ group, whereas instructions to limit them were decreased. The length of hospital stay was shorter among deceased patients only in the ‘late’ group.

Table 3 shows the results of the multiple logistic regression analysis with hospital release status (discharged alive/deceased) as the dependent variable and other study items as explanatory variables. Variables positively associated with being discharged alive were: age (≥85 years), sex (female), early and mid-timing for DNR issuance, diagnosis (non-cancer), internal medicine department, and a shorter hospital stay. Implementation of invasive LST was negatively associated with being discharged alive. To calculate the adjusted odds ratio (AOR) for each item, multiple logistic regression analysis was performed with hospital release status (being discharged alive) as the objective variable and all other study items as explanatory variables. Some of the explanatory variables are continuous variables, and others are categorical variables, so direct comparison cannot be performed, but it can be interpreted that a variable is positively associated with being discharged alive in case of its AOR exceeds 1. The largest AOR was in the early DNR group.

**Table 3:** A patient’s likelihood to be discharged alive as associated with other study variables (multiple logistic regression analysis)

	AOR	95% CI		*p* value
Age, years	1.018	(1.009,	1.027)	< 0.001
Female patients	1.336	(1.120,	1.595)	0.001
Timing of DNR order: Early	13.727	(9.253,	20.366)	< 0.001
Timing of DNR order: Mid-term	6.394	(4.267,	9.581)	< 0.001
Non-cancer patients	3.402	(2.758,	4.197)	< 0.001
Internal medicine patients	1.629	(1.248,	2.127)	< 0.001
Invasive LST	0.358	(0.295,	0.436)	< 0.001
Restrictions on invasive LST	0.956	(0.793,	1.152)	0.634
Hospital length of stay, days	1.005	(1.004,	1.007)	< 0.001

DNR: do not resuscitate, LST: life-sustaining treatment, AOR: adjusted odds ratio, CI: confidence interval. All explanatory variables were adjusted.

Hosmer and Lemeshow goodness-of-fit test (χ^2^ = 14.7831, df = 8, *p* = 0.0635)

### Additional statistical analysis

With regard to the timing of DNR order issuance, no significant differences were observed in separate logistic regression analyses for days 1–5. That is, the timing was not an influential factor during these days. In the ‘late’ and ‘early’ groups, 222 patients were re-categorized from ‘late’ to ‘early’ when early priority criteria were used. However, separate logistic regression analyses for both ‘late’ and ‘early’ groups showed identical results, suggesting that the priority criteria had no effect.

## Discussion

### Principal findings

1. Characteristics of patients discharged alive as compared to deceased patients, particularly with regard to the timing of DNR order issuance

One variable associated with the highest AOR (> 13.7) for patients discharged alive was an early timing of DNR order issuance. In other words, at the time of hospital admission, one group of patients with early DNR issuance who ultimately were discharged alive could be in less severe states, and thus regarded as “uncertain in terms of medical futility but needing some sort of code status.” Our findings are consistent with a previous report from the United States [6].

In addition to the timing of DNR order issuance, those discharged alive were more likely to be elderly, female, non-cancer, and internal medicine patients. Moreover, these patients were less likely to undergo invasive LST.

2) Rates of DNR order issuance in Japan

DNR orders have taken root in Japan; in fact, 45% of all patients who left the hospital deceased had a DNR order. This percentage was lower than that reported by previous studies (92% of patients in the internal medicine department and 56% of surgical patients in Belgium [7], and 65% of all patients in Germany [8]. Moreover, 41% of patients with DNR orders were discharged alive, which was somewhat lower than results from previous studies (51% in the UK [9] and 80% in Australia [10]), suggesting that Japan is no different from other countries in terms of the propensity of DNR orders being liberally issued.

### Strengths of the study

One strength of this study is its methodological robustness compared to previous studies. By analyzing DNR orders issued at 1, 2, 3, 4, and 5 days after admission, we were able to demonstrate that the exact day of DNR order issuance is not critical when the order is issued within 5 days of admission. This effectively minimizes the arbitrariness of analyses based on the day of DNR order issuance. Many previous studies have used ‘within 24 hours after submission of the DNR order’ as a criterion, but this is somewhat problematic. For example, for a patient admitted at midnight, it would be difficult to determine an accurate time for when the DNR order was issued. If this criterion is used, patients admitted at 23:00 and issued a DNR order at 2:00 would be categorized into the 2 days (after admission) group. To avoid this uncertainty, we chose to avoid the criterion of “within 24 hours.”

Another strength is that, although the tendency of elderly patients [11], female patients [12], and cases assigned to an internal medicine physician [13] receiving more DNR orders has been reported, we analyzed all of these factors simultaneously and adjusted for all variables.

Finally, we focused on patients with DNR orders who were discharged alive. One of the most striking findings was that non-cancer patients who were discharged alive were more likely to have been issued a DNR order early after admission. We speculate that these early DNR orders issued to elderly non-cancer patients reflect the desire of physicians, patients, and families to stop all other invasive LSTs.

### Limitations of the study

Our study also has some limitations. First, we adopted a retrospective observational study design that used medical records from a single institution. Thus, our findings are not generalizable. Further studies will be needed to determine how representative our data are, even within Japan.

Second, some of the methodologies used to obtain the present findings might have resulted in lower accuracy. Medical charts are filled out by physicians, with possible distortion, false recognition, erroneous omission, and misclassification. Moreover, we treated each hospitalization by a single patient as a separate case. Thus, some patients may have discussed DNR orders during a previous hospitalization, which could have led to an earlier DNR order. This situation could differ from that of a patient who discussed a DNR order for the first time upon hospitalization.

Third, data on LST performance and restrictions on LST were collected without regard for whether they occurred before or after the DNR order was issued, i.e., these data were collected throughout the entire study period.

Fourth, we did not consider the possibility that treatment restrictions associated with DNR orders led to the death of non-terminal patients.

Finally, although a portion of patients who were discharged alive might have died immediately after discharge, this could not be examined with the present study design. Thus, our findings do not offer sufficient clarity with regard to chronology, or address the cause and effect relationship between DNR order issuance and outcome or treatment restrictions. A prospective study will be needed to address this.

### Significance of the study

This study has shown that, in addition to the timing of DNR order issuance, those who were discharged alive were more likely to be elderly, female, non-cancer, and internal medicine patients. What are the implications of these findings?

Our findings suggest that patients issued late DNR orders were younger, and that many of these patients died during hospitalization. Many of the ‘late DNR orders’ were those issued during the terminal stage of cancer in patients with poor prognoses, for example, when death is imminent and treatment is absolutely futile.

Significantly more female patients who were discharged alive were issued early DNR orders, which is consistent with previous reports [12]. We surmise that these findings are generalizable. A large proportion of the elderly population is female, so the effects of age may confound the result. Thus, in order to adjust for the effects of age, we used age as both continuous and categorical variables. This analysis revealed no difference in results. Nonetheless, careful interpretation is necessary since we were able to adjust only for variables available in medical records.

Our finding that patients discharged alive were significantly more common among patients in internal medicine departments is consistent with results from a previous study [14], which found that this is not necessarily a characteristic of the patient per se, but rather reflective of differences in attitude toward invasive LST from that of surgical departments. In that study, the authors speculated that it might be indicative of a surgeon’s hesitancy to forgo curative measures and embrace palliative care, which could delay DNR order issuance.

### Unanswered questions

As discussed above, non-cancer patients who were discharged alive were more likely to be issued DNR orders early or mid-timing after admission. We speculate that, for elderly non-cancer patients, the desires of physicians, patients, and families to stop all other invasive LSTs come into play. Studies to examine how this practice of DNR benefits the patients’ best interest would be informative.

We also found that DNR orders issued to patients who died during hospitalization were less likely to have been ordered soon after admission (i.e., the ‘late’ group). Unfortunately, this could not be examined further given the lack of data. We speculate that physicians may feel that 1) because cancer patients have a poor prognosis, it is not necessary to consult the patients or their families until death is imminent, and/or 2) physicians do not want to bear the bad news immediately after admission when a relationship of trust has yet to be made between the physician, patient, and family.

We further speculate that resource allocation may contribute to the timing of DNR order issuance. Given the financial burden that accompanies high medical care expenses, physicians shouldering the role of gatekeepers may very well be operating with a strong emotional bias toward avoiding excessive medical care [15].

## Conclusions

By focusing on subjects issued a DNR order but then discharged alive from the hospital, we were able to demonstrate that those subjects, for whom DNR orders tended to be issued early after admission, comprised relatively higher proportions of patients who were either elderly, female, non-cancer, or from the internal medicine department. Our main purpose is to promote the participation of patients in treatment decision-making, and to respect the wishes of patients at the end of life, including decisions regarding DNR. These data would be fundamental for a further investigation to elucidate the reasons for why these characteristics (including socio-economic and cultural factors) of DNR orders are evident among those who are ultimately discharged alive.

## Supplementary information

**Additional file 1.** Dataset of the study. Anonymous data set analyzed.

## Data Availability

All datasets (with anonymity) were attached in an Excel file.

## Abbreviations

DNR: Do not resuscitate
LST: Life sustaining treatment
AOR: Adjusted odds ratio
CI: Confidence interval

## References

[1] Guidelines for the Appropriate Use of Do-Not-Resuscitate Orders. JAMA J Am Med Assoc. American Medical Association; 1991;265:1868. 10.1001/jama.1991.03460140096034 PMID: 2005737. 2005737

[2] Fritz ZB, Barclay SI. Patients' resuscitation preferences in context: lessons from POLST. Resuscitation. 2014; 85(4):444–5. 10.1016/j.resuscitation.2014.01.016. PMID: 24486795. 10.1016/j.resuscitation.2014.01.016 24486795

[3] Fromme EK, Zive D, Schmidt TA, Olszewski E, Tolle SW. POLST Registry do-not-resuscitate orders and other patient treatment preferences. JAMA. 2012; 307(1):34–5. 10.1001/jama.2011.1956 PMID: 22215159. 10.1001/jama.2011.1956 22215159

[4] Hemphill JC, Newman J, Zhao S, Johnston SC. Hospital usage of early do-not-resuscitate orders and outcome after intracerebral hemorrhage. Stroke. 2004;35:1130–4. 10.1161/01.STR.0000125858.71051.ca. PMID:15044768. 10.1161/01.STR.0000125858.71051.ca 15044768

[5] Jackson EA, Yarzebski JL, Goldberg RJ, Wheeler B, Gurwitz JH, Lessard DM, et al. Do-Not-Resuscitate Orders in Patients Hospitalized With Acute Myocardial Infarction. Arch Intern Med. 2004;164:776. 10.1001/archinte.164.7.776. PMID:15078648.. 10.1001/archinte.164.7.776 15078648

[6] Torke AM, Sachs GA, Helft PR, Petronio S, Purnell C, Hui S, et al. Timing of do-not-resuscitate orders for hospitalized older adults who require a surrogate decision-maker. J Am Geriatr Soc. 2011;59:1326–31. 10.1111/j.1532-5415.2011.03480.x. PMID:21732923.. 10.1111/j.1532-5415.2011.03480.x PMC3167071 21732923

[7] Piers RD, Benoit DD, Schrauwen WJ, Van Den Noortgate NJ. DO-not-resuscitate decisions in a large tertiary hospital: Differences between wards and results of a hospital-wide intervention. Acta Clin Belg. 2011;66:116–22. 10.2143/ACB.66.2.2062529. PMID:21630608.. 10.2143/ACB.66.2.2062529 21630608

[8] Becker G, Sarhatlic R, Olschewski M, Xander C, Momm F, Blum HE. End-of-Life Care in Hospital: Current Practice and Potentials for Improvement. J Pain Symptom Manage. 2007;33:711–9. 10.1016/j.jpainsymman.2006.09.030. PMID:17531911.. 10.1016/j.jpainsymman.2006.09.030 17531911

[9] Fritz ZB, Heywood RM, Moffat SC, Bradshaw LE, Fuld JP. Characteristics and outcome of patients with DNACPR orders in an acute hospital; An observational study. Resuscitation. European Resuscitation Council, American Heart Association, Inc., and International Liaison Committee on Resuscitation.~Published by Elsevier Ireland Ltd; 2014;85:104–8. https://doi.org/10.1016/j.resuscitation.2013.08.012. PMID:23994803. 10.1016/j.resuscitation.2013.08.012 23994803

[10] Li JYZ, Yong TY, Hakendorf P, Ben-Tovim D, Thompson CH. The survival of patients with not-for-resuscitation orders. Qjm. 2013;106:903–7. 10.1093/qjmed/hct120. PMID:23676415. 10.1093/qjmed/hct120 23676415

[11] Hakim RB, Teno JM, Jr FEH, Knaus WA, Wenger N, Phillips RS, et al. Factors Associated with Do-Not-Resuscitate Orders: Patients’ Preferences, Prognoses, and Physicians’ Judgments. Ann Intern Med. 1996; 1996;125:284–93.. Available: http://annals.org/aim/fullarticle/709878/factors-associated-do-resuscitate-orders-patients-preferences-prognoses-physicians-judgments. PMID: 8678391. 10.7326/0003-4819-125-4-199608150-00005 8678391

[12] Bardach N, Zhao S, Pantilat S, Johnston SC. Adjustment for do-not-resuscitate orders reverses the apparent in-hospital mortality advantage for minorities. Am J Med. 2005;118:400–8. 10.1016/j.amjmed.2005.01.008. PMID:15808138. 10.1016/j.amjmed.2005.01.008 15808138

[13] Morrell ED, Brown BP, Qi R, Drabiak K, Helft PR. The do-not-resuscitate order: Associations with advance directives, physician specialty and documentation of discussion 15 years after the Patient Self-Determination Act. J Med Ethics. 2008;34:642–7. 10.1136/jme.2007.022517. PMID: 18757631. 10.1136/jme.2007.022517 18757631

[14] Kelley AS, Gold HT, Roach KW, Fins JJ. Differential Medical and Surgical House Staff Involvement in End-of-Life Decisions: A Retrospective Chart Review. J Pain Symptom Manage. 2006;32:110–7. 10.1016/j.jpainsymman.2006.02.009. PMID: 16877178. 10.1016/j.jpainsymman.2006.02.009 16877178

[15] Cooper AB, Sibbald R, Scales DC, Rozmovits L, Sinuff T. Scarcity: The Context of Rationing in an Ontario Icu*. Crit Care Med. 2013;41:1476–82. 10.1097/ccm.0b013e31827cab6a. PMID: 23474676. 10.1097/CCM.0b013e31827cab6a 23474676

